# Chronic Quetiapine Administration Has a Therapeutic Effect on Anxiety and Depression‐Like Behavior in a Chronic Unpredictable Mild Stress (CUMS) Rat Model

**DOI:** 10.1002/brb3.70755

**Published:** 2025-08-21

**Authors:** Esra Komanovalı, Burcu Çevreli, Öznur Özge Özcan

**Affiliations:** ^1^ Molecular Biology, Institute of Science Üsküdar University İstanbul Turkey; ^2^ Department of Physiology, Faculty of Medicine Üsküdar University İstanbul Turkey; ^3^ Molecular Biology and Genetics, Faculty of Engineering and Natural Sciences Üsküdar University Istanbul Turkey

## Abstract

**Background:**

The mechanisms underlying quetiapine's effects on anxiety‐ and depression‐like behaviors induced by chronic unpredictable mild stress (CUMS) remain unclear. This study aimed to evaluate the therapeutic potential of quetiapine in a rat model of CUMS.

**Materials and Methods:**

In the first experiment, 40 adult female Wistar albino rats were randomly assigned to either a control group (non‐stressed, *n* = 20) or a chronic unpredictable mild stress (CUMS) group (*n* = 20) for 28 consecutive days. To evaluate stress‐related behavioral changes, the sucrose preference test (SPT), fecal boli count, and elevated plus maze (EPM) test were conducted on days 29 and 30. In the second experiment, on day 30, animals from both initial groups were further divided into four experimental groups (*n* = 10 per group): (1) Control (1 mL saline, i.p., for 30 days), (2) Quetiapine (QET; 10 mg/kg/day, i.p., for 30 days), (3) CUMS only (no treatment), and (4) CUMS + QET (10 mg/kg/day, i.p., for 30 days). Following treatment, behavioral assessments (SPT, forced swim test (FST), elevated plus maze test (EPM), and fecal boli count) were conducted on days 61, 62, and 63.

**Results:**

CUMS induced significant anxiety‐ and depression‐like behaviors. Chronic quetiapine administration (10 mg/kg/day) reversed these behavioral deficits, suggesting antidepressant and anxiolytic effects.

**Conclusion:**

Quetiapine alleviates CUMS‐induced anxiety and depression‐like behaviors, supporting its potential as both monotherapy and adjunctive treatment in stress‐related disorders. Further research should explore sex differences, dose‐response effects, biochemical or molecular markers, and other stress paradigms to optimize clinical relevance.

## Graphical abstract

This article presents the effects of chronic quetiapine administration on anxiety and depression with and without chronic unpredictable mild stress disorder (CUMS) in female adult Wistar albino rats using behavioral analyses. Quetiapine effectively mitigates depression‐ and anxiety‐like behaviors induced by chronic stress, supporting its potential utility both as monotherapy and as an adjunct to existing pharmacotherapies. Further investigations are warranted to assess its efficacy across diverse stress models, sex‐related variations, and dose‐response relationships, with the goal of refining treatment strategies.

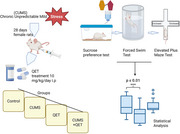

## 1 Introduction

Depression is a widespread condition with significant implications for the physical and mental well‐being of individuals worldwide. It imposes a substantial social burden and is a major factor in suicidal thoughts. Selective serotonin reuptake inhibitors (SSRIs) are frequently used as the primary pharmacological intervention for depression. Nevertheless, extended SSRI use has been linked to the development of adverse effects over time. Additionally, a considerable proportion (30–40%) of individuals with depression do not experience symptom remission with these treatments. Given the rising prevalence of depression, the identification of safer and more effective antidepressant therapies is a critical and pressing concern (Remes et al. 2021). Clinically, depression presents with symptoms such as a persistently low mood, decreased motivation, cognitive dysfunction, and psychomotor retardation (Strekalova et al. 2022). According to data from the World Health Organization, around 350 million individuals globally are affected by depression, and more than 800,000 people die by suicide each year (Caspi et al. 2024). By 2025, depression is projected to rank among the top two leading contributors to disability‐adjusted life years. Although current antidepressant treatments achieve remission in roughly 60% to 80% of cases, only about 30% of patients experience a favorable long‐term prognosis. These statistics highlight the urgent need for innovative therapeutic approaches that can improve patient outcomes more effectively (Aaron et al. 2025). Epidemiological research indicates that women are more susceptible to stress‐related mental health issues compared to men and have approximately double the risk of developing depression (Zhang et al. 2023).

Chronic unpredictable mild stress (CUMS) is frequently recognized as a widely used preclinical model for major depressive disorder (MDD) (Markov and Novosadova 2022). In preclinical research, the CUMS model is the most commonly employed method in rodent studies. It involves exposing animals to a series of varied, unpredictable, and uncontrollable stressors over a period of days or weeks. Originally developed as a model to simulate depression, this model offers a relevant representation of depression by replicating everyday stressors similar to those experienced by humans—a key symptom of depressive disorders as defined in the Diagnostic and Statistical Manual of Mental Disorders, Fourth Edition (DSM‐IV) (Antoniuk et al. 2019). Additionally, depressive‐like behavior triggered by the CUMS model typically responds to chronic, but not acute, antidepressant treatment, which enhances its validity as a realistic model of human depression (Binnetoğlu et al. 2019). CUMS continues to be one of the most extensively used paradigms. Like other stress‐based models, it has been primarily validated in adult rodents; however, there is a notable lack of research involving younger animals and females. While depression is prevalent across all age groups and shows a higher incidence in women, the behavioral alterations linked to quetiapine treatment in such contexts have yet to be fully elucidated.

Quetiapine is uniquely approved by the U.S. Food and Drug Administration (FDA) as a standalone treatment for both manic and depressive episodes in bipolar disorder (Suppes et al.,^2010^ ), as well as an add‐on therapy for major depressive disorder (MDD) (Grolli et al., 2023). Animal studies support its antidepressant‐like properties; for example, it has been shown to reduce anhedonia in rats subjected to CUMS (Burstein and Doron 2018). Quetiapine, like other atypical antipsychotics, has been extensively studied as an add‐on treatment for MDD and treatment‐resistant depression (TRD), leading to its FDA approval for these indications (Ignácio et al. 2017; Zhou et al. 2015). Quetiapine's antidepressant properties are thought to arise, at least in part, from its partial agonist activity at 5‐HT1A receptors and its strong inhibition of the norepinephrine transporter (NET) mediated by its active metabolite, N‐desalkylquetiapine (Jensen et al. 2008; Cross et al. 2016). Considering this information, in this study, we aimed to investigate the antidepressant effects of quetiapine in female rats subjected to the CUMS model. Therefore, in the current study we also evaluated the efficacy of chronic 10 mg/kg/day, i.p. quetiapine administration in female rats in preventing anhedonia, depression, and stress in rats previously exposed to the CUMS protocol.

## 2 Material and Methods

A total of 40 female Wistar albino rats, 8 weeks old and weighing 200–250 g, were used. The animals were kept in constant environmental conditions of 22 ± 2°C, 50 ± 10% relative humidity, and a 12‐h light/12‐h dark cycle throughout the experiment. All rats were fed with standard laboratory chow and had free access to water throughout the experiment. All procedures were approved by the Animal Research Ethics Committee of Üsküdar University (Ü.Ü‐HADYEK, Decision No: 2024–09, October 10 2024) and conducted in accordance with the European Directive 2010/63/EU and national guidelines for the care and use of laboratory animals. Efforts were made to minimize animal discomfort throughout the study.

**FIGURE 1:**
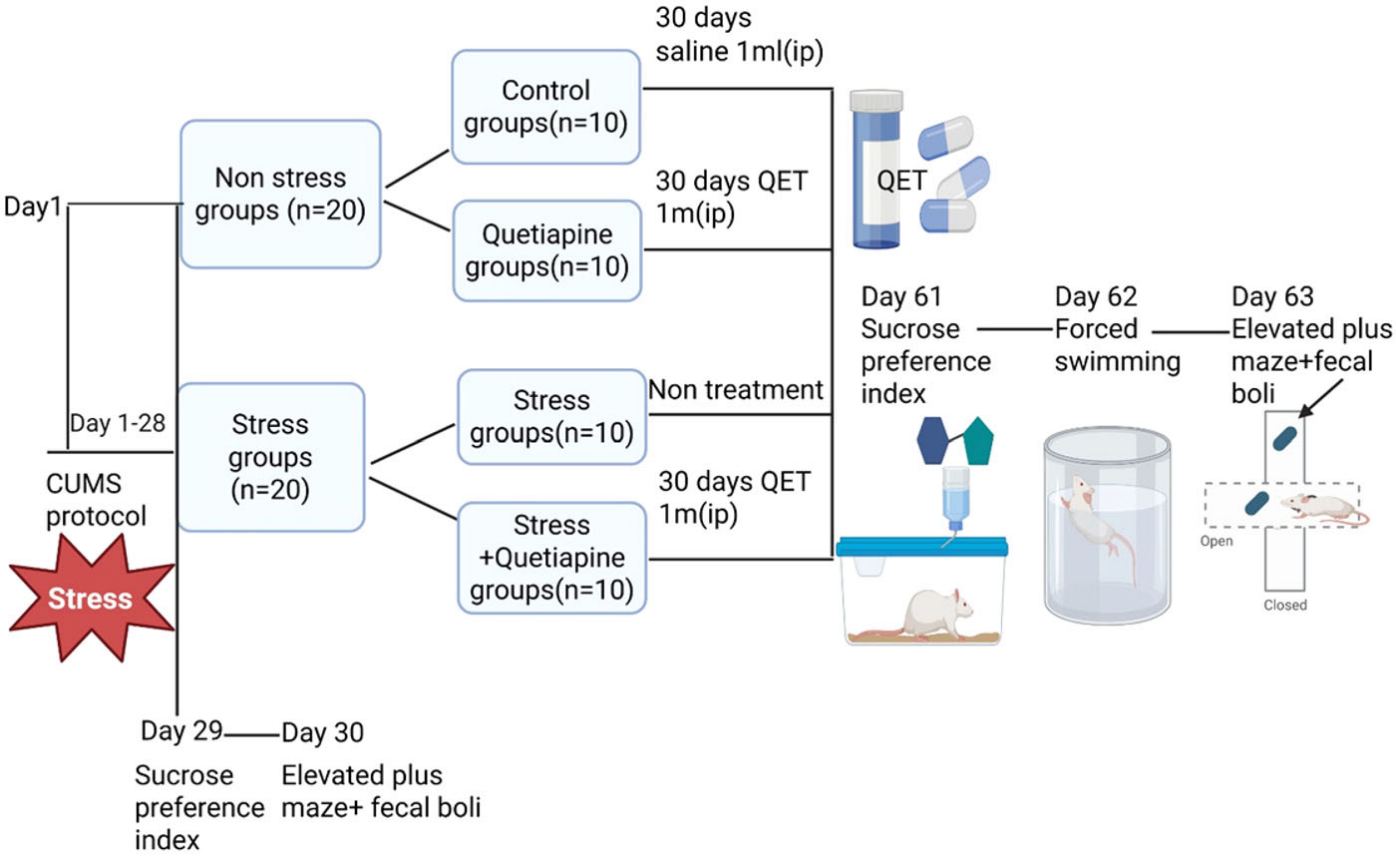
A timeline diagram for experimental design.

In the first experiment, 40 female Wistar albino rats (8 weeks old, 200–250 gr) were divided into two groups with five rats per cage (*n* = 5) in two separate experimental rooms: the stress group that received 28 days of CUMS protocol (CUMS‐total, *n* = 20) and and the control group (non‐stress group, Control‐total, *n* = 20) that received no intervention for 28 days. At the end of 28 days, the sucrose preference test (SPT), fecal boli count, and elevated plus maze test (EPM) were performed on days 29 and 30 to determine the stress response between the groups. As a result of the first experiment, anxiety, depression, and stress parameters of all rats in the stress group were significantly elevated compared to the control group, and accordingly, in the second experiment, 20 rats in the CUMS‐total group were randomly divided into two groups to investigate the effects of quetiapine treatment: a positive control (non‐treatment, CUMS group, *n* = 10) and a group administered intraperitoneal (i.p.) injection of 10 mg/kg/day of quetiapine dissolved in 1 mL of saline for 30 days (CUMS+QET group, *n* = 10). The rats in the control‐total group were also randomly divided into two groups: a negative control received 1 mL saline intraperitoneally for 30 days (control group, *n* = 10), and a group that received intraperitoneal (i.p.) injection of 10 mg/kg/day of quetiapine dissolved in 1 mL of saline for 30 days (QET group, *n* = 10). As a result, in our study, four experimental groups were established: (1) Control (*n* = 10), (2) QET (*n* = 10), (3) CUMS (*n* = 10), and (4) CUMS + QET (*n* = 10). At the end of all treatment protocols, SPT, the forced swimming test (FST), EPM, and fecal boli count were assessed in all groups on days 61, 62, and 63. All experimental designs are shown in Figure 1.

### 2.1 Drugs and Administration

Quetiapine fumarate (Sigma‐Aldrich, USA) was prepared as an injection solution and administered intraperitoneally (i.p.) at a dose of 10 mg/kg at the same time every day throughout the experimental period. Drug administration was performed regularly for a total of 30 days. The pure quetiapine powder was administered via intraperitoneal injection at a dosage of 10 mg/kg/day, dissolved in 1 mL of saline solution. This dosage was selected based on guidelines for converting human doses to appropriate animal doses. The quetiapine (10 mg/kg/day, i.p.) was dissolved in physiological saline solution and administered, following previous research to minimize potential adverse effects during chronic administration (mortality in the experiment) (He et al. 2020). This specific dose and solvent were chosen in accordance with earlier studies (Zhou et al. 2020; Wang et al. 2015). In compliance with OECD Guideline no. 407, the injection volume for animals did not exceed 2 mL per 100 g body weight, with each rat receiving a total injection volume of 1 mL of the drug solution.

### 2.2 CUMS Protocol

During the experimental period, rats were exposed to various stress factors according to the CUMS protocol. Stress applications were conducted daily between 9:00 and 11:00 a.m. for 28 days.

In order to induce depression‐like behaviors in experimental animals, the standard CUMS protocol defined by Garabadu et al. 2019. In short, this protocol, consisting of different stress factors each week, was randomly presented to the rats in both active (dark) and inactive (light) periods. The following stressors were administered during the protocol:

- Paired cage: kept in the same cage with a different rat for 2 h.
- Tilted cage: placed in a cage inclined at a 45° angle for 3 h.
- Food restriction: deprived of food for 18 h, followed by limited food access for 1 h.
- Water restriction: denied access to water for 18 h, followed by exposure to an empty water bottle for 1 h.
- Wet cage: kept in a wet sawdust environment for 21 h (100 g sawdust + 200 mL water).
- Continuous light exposure: exposed to uninterrupted light for 36 h.

Stress factors were randomly applied so that rats could not predict stress, and their adaptation mechanisms were disrupted. While some stressors, such as food and water restriction, may have metabolic consequences, their application was limited and alternated with non‐metabolic stressors to minimize systemic effects. Control group animals were kept under standard laboratory conditions throughout the experimental period and were not exposed to any stress factors. At the beginning of the experiment, rats were acclimated for 1 week.

### 2.3 SPT

The SPT was applied according to a previous study (Liu et al. 2018) to determine anhedonia and depression‐like behavior (reduction in hedonic capacity). The test was performed between 09:00 and 10:00 a.m. in the morning. Animals were deprived of food and water for 20 h before the test and then allowed to consume both water and 1% sucrose solution for 1 h. Sucrose was calculated as the ratio of sucrose solution consumption to total fluid intake. This ratio was determined by the following formula: Sucrose preference index = (sucrose intake/total intake) × 100%.

### 2.4 Forced Swim Test (FST)

The FST test is commonly used to assess depression‐like behavior in rodents. The test protocol was applied according to a previous study (Wang et al. 2021). During the test, each rat was placed in a cylindrical tank made of clear glass (height: 50 cm, diameter: 20 cm) with water at 25°C. The animals were allowed to move freely in the water and were observed for a total of 6 min. During this period, the immobility time (time of remaining motionless), active swimming time, and climbing attempts were recorded. A long immobility time was considered an indicator of depression‐like helplessness behavior. Before the test, the rats were given a 15‐min pre‐adaptation, and the main test was performed the next day.

### 2.5 EPM

The EPM test is a standard paradigm used to determine anxiety‐like behavior. The protocol was applied according to Zhu et al.’s protocol in 2022. The elevated plus maze is a platform 50 cm above the floor, consisting of two open and two closed arms. Rats were placed in the center area of ​​the maze, and the time spent in the open and closed arms, the number of entries into the open arms, and general locomotor activity were recorded for 5 min. Spending less time in the open arms and making fewer entries were considered indicators of anxiety‐like behavior. The room was kept quiet during the test to prevent the animals from being affected by environmental factors. The number of fecal boli was also recorded to understand the anxiety/stress level.

### 2.6 Statistical Analysis

Statistical analysis was performed using GraphPad Prism v10.4.1. We calculated sample sizes of minimum *n* = 10 animals per condition using the previous study of CUMS protocol (Li et al. 2025), *α* = 0.05, and power = 0.80, to detect at least a 25% difference in mean values for treated and control samples using G^×^Power (Faul et al. 2007). A test for normality was performed to select the appropriate statistical method. All data are expressed as the mean ± SD.

Additionally, Student's *t*‐test was applied to measure differences between specific groups. In all experimental groups, significance between groups according to each measurement time was assessed using one‐way analysis of variance (ANOVA). All datasets were assessed for normality using the Shapiro–Wilk test prior to parametric analyses. Fecal boli data met the assumptions of normal distribution (*p* > 0.05), justifying the use of one‐way ANOVA. Bonferroni tests were used as post‐hoc tests to determine which group caused the difference. *p* < 0.05 was considered statistically significant.

## 3 Results

**FIGURE 2:**
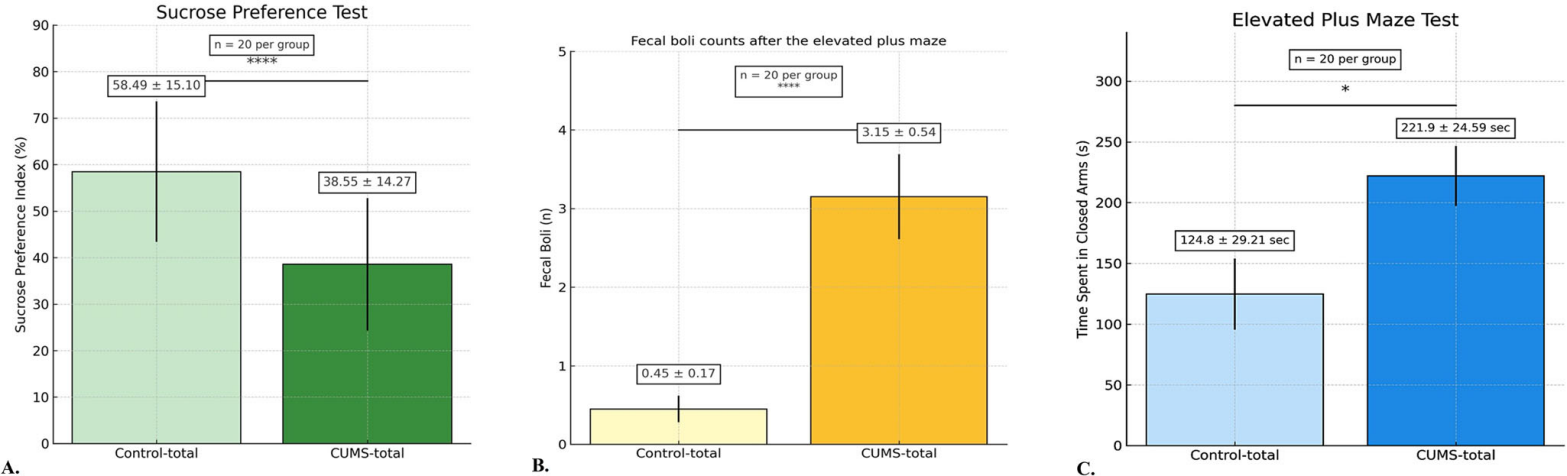
The results of the CUMS (28 days) intervention: **(A)** Results of the sucrose preference test (SPT) and **(B)** Fecal boli counts after the elevated plus maze (EPM) to evaluate stress/anxiety. **C**. Anxiety‐like behaviors in rats according to the time spent in the closed arm of the EPM test. ****p < 0.001, *p < 0.05. Student's *t*‐test followed by post hoc Bonferroni correction (*n* = 20 for each group). Values are expressed as mean ± SD. Numerical values (mean ± SD).

First, the effects of 28 days of CUMS were evaluated in the CUMS‐total group (n = 20). As shown in Figure 2, Student's t‐test was used to compare the mean values of the SPT, fecal boli counts, and time spent in the closed arm of the EPM between the CUMS‐total and Control‐total groups (*n* = 20 each). The sucrose preference index mean of the CUMS‐total group decreased significantly compared to the Control‐total group (in Figure 2A, *p* < 0.001, 95% confidence interval (CI), from ‐27.53 to ‐12.14. *t* = 5.395, df = 19). When the means and standard deviations of the groups were calculated, it was found that the Control‐total group was 58.49 ± 15.10 and the CUMS‐total group was 38.55 ± 14.27. The number of fecal boli was 0.4500 ± 0.1698 for control‐total group and 3.150 ± 0.5442 for CUMS‐total group. The results of fecal boli were shown in Figure 2B. CUMS‐total group had significantly higher fecal boli compared to the control‐total group (*p* = 0.0001, 95% CI from 1.533 to 3.867, *t* = 4,841, df = 19). The average time spent in the closed arm in the EPM, CUMS‐total group increased significantly compared to the Control‐total group (in Figure 2C, *p* = 0.0112, 95% CI from 24.79 to 169.5, *t* = 2.810, df = 19). When the means and standard deviations of the groups were calculated, it was found that the Control‐total group was 124.8 ± 29.21 and the CUMS‐total group was 221.9 ± 24.59.

**FIGURE 3:**
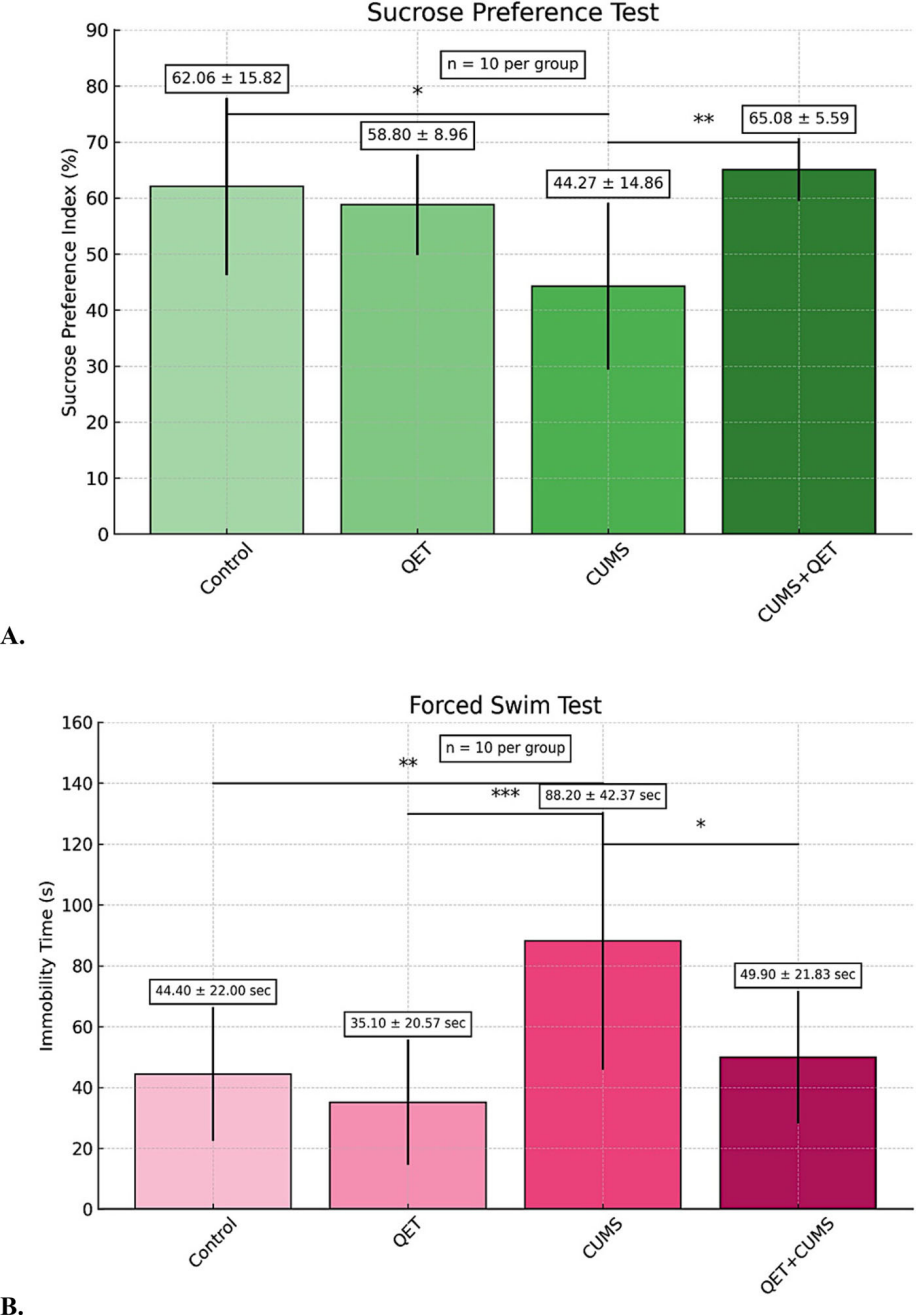
**(A)** Effect of chronic quetiapine administration (10 mg/kg/day, i.p., for 30 days) on CUMS‐induced anhedonia and depression‐like behavior in female Wistar albino rats. Effects of CUMS and quetiapine (30 days, 10 mg/kg) intervention on the sucrose preference test. These results indicate that chronic stress significantly reduces sucrose preference, suggesting anhedonia. Chronic quetiapine treatment (10 mg/kg/day, i.p., 30 days) effectively reverses this effect and restores reward sensitivity and **(B)** 30‐day quetiapine treatment (10 mg/kg/day, i.p.) protects against depression‐like behavior induced by repeated forced swim stress (FST). The results show that chronic stress increases immobility time, indicating behavioral despair, while chronic quetiapine administration significantly reduces this effect, suggesting an antidepressant‐like action. ***p < 0.005, **p < 0.01, and *p < 0.05. One‐way ANOVA followed by post hoc Bonferroni test (*n* = 10 for each group). Values are expressed as mean ± SD. Numerical values (mean ± SD).

To assess group differences in the SPT results following chronic quetiapine administration, a one‐way ANOVA was conducted, revealing a significant effect of treatment (F (3, 36) = 5.835, *p* = 0.0023). When the means and standard deviations of the groups were calculated, it was found that the control group was 62.06 ± 15.82, the QET group was 58.80 ± 8.959, the CUMS group was 44.27 ± 14.86, and the CUMS + QET group was 65.08 ± 5.588. The sucrose preference of the CUMS group was significantly lower compared to the control group (*p* = 0.0113, 95% Confidence Interval (CI) from 3.252 to 32.32, *t* = 4.661). The mean sucrose preference of the CUMS+QET group was significantly increased compared to the CUMS group (*p* = 0.0025, 95% CI, from ‐35.34 to ‐6.273, *t* = 5.453). The findings indicate that chronic stress reduces sucrose preference, indicating depression‐like behavior. Chronic quetiapine administration (10 mg/kg/day, i.p., 30 days) may reverse these effects and attenuate depression‐like symptoms induced by chronic stress (in Figure 3A).

As shown in Figure 3B, in order to find the difference between the groups in the immobility duration means in the FST results and to find a statistically significant difference, especially for chronic quetiapine administration, a one‐way ANOVA was revealed, and a significant difference was found between the groups (F (3, 36) = 6.860, *p* = 0.0009). When the means and standard deviations of the groups were calculated, it was found that the control group was 44.40 ± 22.00, the QET group was 35.10 ± 20.57, the CUMS group was 88.20 ± 42.37, and the CUMS + QET group was 49.90 ± 21.83. The immobility duration mean of the CUMS group increased significantly compared to the control group (*p* = 0.0081, 95% CI, from ‐79.00 to ‐8.603, *t* = 3.474). The mean immobility time of the CUMS + QET group decreased significantly compared to the CUMS group (*p* = 0.0265, 95% CI, from ‐3.103 to 73.50, *t* = 3.038). Interestingly, the mean immobility time of the QET group also decreased significantly compared to the CUMS group (*p* = 0.0010, 95% CI, from ‐88.30 to ‐17.90, t = 4.212). Findings showed that while chronic stress causes depression‐like behavior by increasing the immobility time, chronic quetiapine administration (10 mg/kg/day, i.p., 30 days) can reverse these effects and reduce the symptoms of depression caused by chronic stress. (in Figure 3B).

**FIGURE 4:**
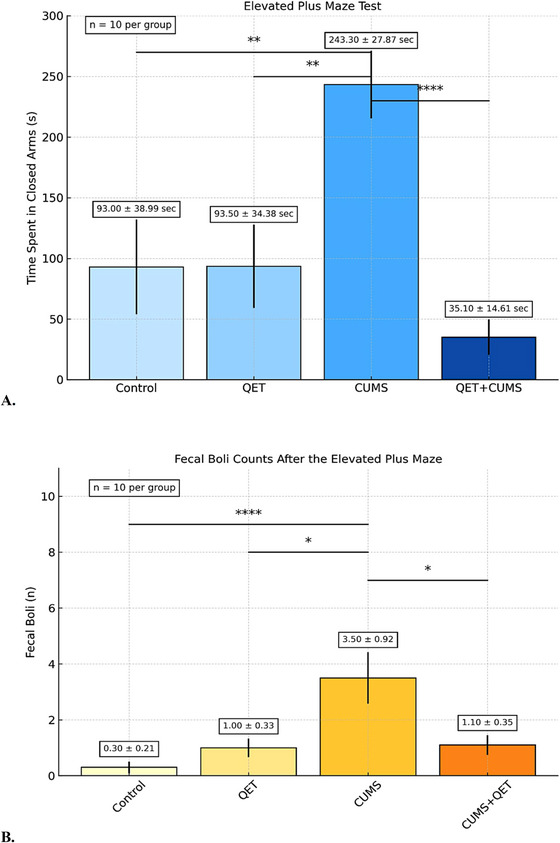
**(A)** Effects of chronic quetiapine treatment (10 mg/kg/day, i.p., for 30 days) on anxiety‐like behavior andfecal boli in female Wistar albino rats following CUMS exposure. Effects of chronic stress and chronic quetiapine (30 days, 10 mg/kg) intervention on anxiety‐like behaviors in rats, based on time spent in the closed arm of the EPM. The findings show that chronic stress significantly increases time spent in the closed arm, indicating heightened anxiety‐like behavior. Chronic quetiapine administration (10 mg/kg/day, i.p., 30 days) mitigates these effects and reduces anxiety‐related responses in the EPM and **(B)** Fecal boli count following the EPM test as a physiological indicator of stress/anxiety: The number of fecal boli significantly increased in the CUMS group, further supporting its anxiogenic effects. This increase was reversed by chronic quetiapine administration, reinforcing its anxiolytic potential. ****p < 0.001, *** *p* < 0.005, **p < 0.01, and *p < 0.05. One‐way ANOVA followed by post hoc Bonferroni test (*n* = 10 per group). Values are expressed as mean ± SD. Numerical values (mean ± SD).

As shown in Figure 4A, in order to find the difference between the groups in the average time spent in the closed arm in the EPM results and to find a statistically significant difference, especially for chronic quetiapine administration, one‐way ANOVA was revealed, and a significant difference was found between the groups (F (3, 36) = 9.448, *p* <0.0001). When the means and standard deviations of the groups were calculated, it was found that the control group was 93.00 ± 38.99, the QET group was 93.50±34.38, the CUMS group was 243.3 ± 27.87 and the CUMS + QET group was 35.10 ±14.61. The mean time spent in the closed arm in the CUMS group was found to be significantly higher than in the control group (*p* = 0.0076, 95% CI from ‐270.3 to ‐30.35, *t* = 3.498). It was observed that chronic stress increased anxiety levels in the EPM, and rats preferred to stay in the closed arm more. The mean time spent in the closed arm in the CUMS + QET group was found to be significantly lower than in the CUMS group (*p* = 0.0001, CI, from 88.61 to 327.8, *t* = 4.846). Therefore, while chronic stress intervention caused anxiety‐like behavior, findings showed that chronic quetiapine administration (10 mg/kg/day, i.p.) could reverse these effects and reduce anxiety symptoms caused by chronic stress.

The number of fecal boli was 0.3000± 0.2134 for control group, 1.000± 0.3333 for QET group, 3,500± 0,9220 for CUMS group, 1.100 ± 0.3480 for CUMS+QET group. The results of fecal boli were shown in Figure 4B. The differences between the groups for the fecal boli after the EPM test were significant (*p* = 0.0009, F (3, 36)  =  6.913). Fecal boli were counted to determine stress‐/anxiety‐like behavior after EPM test completion, and the CUMS group had significantly higher fecal boli compared to the Control group (*p* = 0.0008, *t* = 4,261), the QET group (*p* = 0.0121, *t* = 3.329), and CUMS + QET group (*p* = 0.0174, *t* = 3.196).

## 4 Discussion

In our study, 28‐day CUMS exposure led to increased anxiety‐like behaviors (EPM), depression‐like behaviors (FST), elevated stress markers (fecal boli), and anhedonia (SPT). The present study provides the first evidence that chronic quetiapine administration is an important modulator in the regulation of anxiety‐ and depression‐like behaviors after the CUMS protocol. Recommended doses of quetiapine as an antipsychotic agent range from 150 to 750 mg/day, depending on individual clinical response and tolerability. Therefore, the quetiapine dose used in the current study is at the conventional (10 mg/kg/day) end of the “therapeutic window.” Kapur et al. (2003) reported that the lowest plasma concentrations of quetiapine in rats using the injection approach were approximately 50–100 times lower than those seen in patients. This results in undetectable D2 receptor occupancy levels at clinically comparable quetiapine doses in rodents. However, compared to dopamine D2 receptors, quetiapine has greater affinity for adrenergic α1 (100‐fold), α2 (10‐fold), histamine H1 (50‐fold), and serotonin 2A (25‐fold) receptors. Therefore, the above considerations regarding the optimum preclinical dosage of quetiapine are probably limited to dopamine D2 receptors and, consequently, the antipsychotic effect of the drug. In any case, similar equivalent occupancy studies in humans and animals should be performed to gain more insight into receptor systems other than dopamine D2 receptors. The dose of 10 mg/kg quetiapine was selected based on previous preclinical studies demonstrating its efficacy in modulating depression‐ and anxiety‐like behaviors in rodent models without inducing significant locomotor impairment or sedation (Hashimoto et al. 2007; Bai et al. 2014). Importantly, this dose is pharmacokinetically relevant, as it achieves central nervous system (CNS) concentrations sufficient to occupy key receptors implicated in mood regulation, including 5‐HT1A, D2, and α2‐adrenergic receptors (Schotte et al. 1996; Raggi et al. 2004). Furthermore, its active metabolite, N‐desalkylquetiapine, contributes to the inhibition of the norepinephrine transporter (NET), which is associated with its antidepressant‐like effects (Jensen et al. 2008). Thus, 10 mg/kg represents a clinically relevant and translationally appropriate dose in rats, aligning with both behavioral efficacy and receptor occupancy data.

Our findings suggest that the reduction in sucrose preference induced by chronic CUMS can be reversed by chronic quetiapine treatment. Under our experimental conditions, quetiapine appears to prevent the development of anhedonia in rats exposed to the CUMS protocol. Following 4 weeks of CUMS, rats treated with 10 mg/kg/day quetiapine show significantly higher sucrose intake than rats subjected to CUMS alone. Similar results were observed in FST and EPM tests. In our study, a 4‐week CUMS intervention in rats increased anxiety‐like behavior according to EPM test results, depression‐like behavior according to FST results, anxiety and stress levels according to fecal boli results, and anhedonia and depression‐like behaviors according to SPT results. The SPT serves as a highly sensitive behavioral test for detecting core depressive phenotypes, particularly anhedonia—a hallmark of impaired reward processing. Under baseline conditions, healthy rodents display a robust preference for sweet solutions. In a study by Shao et al. (2015), male Sprague‐Dawley rats subjected to a 14‐day CUMS protocol demonstrated SPT values exceeding 70% in control groups, suggesting stable hedonic capacity under non‐stress conditions. Berrio et al. (2024) further corroborated this pattern in a systematic review and meta‐analysis, reporting consistently elevated SPT values in male Sprague Dawley controls across CUMS exposures of 2 to 6 weeks. The pooled standardized mean difference (SMD) for sucrose preference was −1.48 (95% CI: ‐1.65 to ‐1.30), underscoring the magnitude of stress‐induced reductions in reward sensitivity and the robustness of anhedonia as a behavioral endpoint. Zhou et al. (2021) similarly reported SPT values above 80% in various botanical treatment models. Collectively, these convergent datasets reinforce the reproducibility and translational relevance of SPT in modeling stress‐induced anhedonia. In our study, female Wistar albino rats in the control group exhibited a sucrose preference rate of 62.06 ± 15.82%, aligning with previously reported baseline values. These findings support the validity of our control conditions and indicate preserved reward sensitivity in the absence of stress exposure. In the stress group, sucrose preference declined to 44.27 ± 14.86%, consistent with prior reports and underscoring the reliability of the applied stress model. Notably, to our knowledge, no prior studies have evaluated sucrose preference in non‐stressed animals following chronic quetiapine administration. Our data indicate that 10 mg/kg/day of quetiapine for 30 days does not alter hedonic processing in unstressed animals. Wang et al. (2013) found that while fluoxetine alone did not normalize SPT values after 4 weeks of CMS, adjunctive quetiapine maintained sucrose preference above 60% and promoted hippocampal neurogenesis. Özcan et al. (2024) also reported antidepressant‐like behavioral restoration following quetiapine administration in stress‐relevant sleep deprivation models. Similarly, Chen et al. (2015) found that a 7‐day combined treatment with rTMS and quetiapine reversed anhedonia and promoted BDNF/ERK pathway activation. Collectively, this supports the dual role of quetiapine in symptom alleviation and neuroplastic modulation under chronic stress conditions. In our study, female Wistar albino rats treated with quetiapine (10 mg/kg/day for 30 days) exhibited a sucrose preference of 65.08 ± 5.59%, consistent with prior observations. These data indicate that chronic quetiapine exposure does not negatively impact hedonic processing and may confer protective effects against the emergence of anhedonia. However, the literature on quetiapine's long‐term effects in chronic stress paradigms remains limited, underscoring the need for further investigation into its mechanistic actions on anhedonia. While quetiapine is emerging as a promising agent in treatment‐resistant depression and anxiety, comprehensive data addressing its sustained efficacy under chronic stress conditions remain scarce.

Broadly, existing data suggest that first‐generation antipsychotics exert limited or even detrimental effects on anhedonia and depression‐like behaviors, whereas second‐generation agents, including quetiapine and haloperidol, demonstrate superior antidepressant efficacy. Both agents have been shown to improve sucrose preference under chronic stress, supporting their therapeutic potential. Our findings are consistent with this body of evidence and further underscore quetiapine's utility in chronic stress models. In addition to SPT, the FST is widely employed to evaluate depressive‐like behaviors and pharmacological interventions in preclinical stress paradigms. In non‐stressed female Wistar albino rats, FST immobility times typically remain within a narrow baseline range. Zhang et al. (2018) reported baseline immobility durations of ∼30 s in controls, whereas exposure to a 3‐week CUMS protocol in 180–220 g female Sprague‐Dawley rats resulted in immobility times approaching 120 s. Similarly, Mishra et al. (2020) demonstrated that 2‐month‐old male C57BL/6J mice (23–27 g) subjected to a 21‐day CUMS protocol exhibited significantly elevated immobility times (146.1 ± 60.8 sec) compared to controls (29.9 ± 19.3 sec). Pei et al. (2025) further reported FST immobility durations of ∼150 s following a 6‐week CUMS exposure in 5–6‐week‐old male Sprague‐Dawley rats (170–200 g), while control animals exhibited immobility around 50 s. Collectively, these studies highlight the robust induction of behavioral despair following chronic stress, while also emphasizing the potential influence of strain, age, sex differences, and environmental variables on behavioral outcomes. In a complementary study, Agarwal et al. (2021) reported that 5‐week‐old male Wistar rats (100–120 g) exposed to an 8‐week CUMS protocol exhibited immobility durations of ∼100 s, compared to <50 s in controls, further supporting the cumulative impact of prolonged stress exposure. In our study, FST immobility durations averaged 44.40 ± 22.00 s in controls and increased to 88.20 ± 42.37 s in the stress group, aligning with previous reports, though the elevated standard deviation observed in the stress group warrants further investigation. Our findings indicate notable inter‐individual variability in stress susceptibility within the experimental cohort, reflecting heterogeneous behavioral responses to chronic stress exposure. While elevated mean immobility times confirm the robust impact of stress, the similarity of these results to those observed in longer‐duration protocols may reflect heightened stress sensitivity in female rats. Supporting this, Wang et al. (2013) demonstrated that after a 4‐week CUMS protocol in Sprague‐Dawley rats (220–250 g), fluoxetine (10 mg/kg/day) alone maintained immobility times above 120 s in the FST, whereas adjunctive quetiapine reduced immobility durations below 120 s, suggesting a synergistic antidepressant effect. Grolli et al. (2023) further linked CUMS‐induced depression‐like behaviors to altered oxidative balance and systemic inflammation. In their study, quetiapine administered at 20 mg/kg significantly attenuated immobility in the FST and exerted anti‐inflammatory effects, implicating modulation of oxidative stress and inflammatory pathways in its mechanism of action. Although higher doses may enhance efficacy, they also increase the risk of sedation. In contrast, the 10 mg/kg dose employed in our study achieved significant reductions in immobility without sedative side effects, providing optimal therapeutic benefit with minimized adverse risk. These findings support the behavioral efficacy of antipsychotics in alleviating stress‐induced depression‐like behaviors, with outcomes strongly dependent on dose, treatment duration, and model‐specific parameters.

In our experiment, female Wistar albino rats exposed to a 28‐day CUMS protocol and subsequently treated with quetiapine (10 mg/kg/day for 30 days) exhibited significantly reduced immobility times (49.90 ± 21.83 s) compared to the untreated stress group (88.20 ± 42.37 s), demonstrating the antidepressant‐like effect of quetiapine in mitigating behavioral despair under chronic stress. However, despite these promising results, studies evaluating quetiapine's effects on depression‐like behaviors in chronic stress models—particularly those employing FST paradigms—remain limited in the literature. In parallel, anxiety‐like behaviors were assessed using the EPM, a validated and highly sensitive test widely utilized to evaluate anxiety phenotypes in rodent models. In this paradigm, avoidance of open arms and preference for enclosed arms is interpreted as an index of heightened anxiety. Tekşen et al. (2024) reported that female Sprague‐Dawley rats (200–250 g) subjected to a 20‐days CUMS protocol spent over 300 s in the closed arms, significantly exceeding control values and indicating anxiety‐like responses. Similarly, Youssef et al. (2022) observed closed‐arm times exceeding 200 s in male Wistar rats (115–135 g) after a 2‐week protocol, again significantly elevated relative to controls. In our study, non‐stressed female Wistar albino rats exhibited closed‐arm times of 93.00 ± 38.99 s, consistent with normative anxiety behavior observed in unstressed controls. Chronic stress exposure significantly increased closed‐arm times to 243.30 ± 27.87 s, reflecting the anxiogenic effects of prolonged stress and the resultant avoidance of open spaces. While CUMS models are frequently utilized to investigate the efficacy of antidepressant interventions, comparatively fewer studies have addressed the anxiolytic potential of antipsychotic agents in these paradigms, highlighting a critical gap in the urrent literature. Kingir et al. (2023) investigated the anxiolytic effects of ketamine in adult male Wistar rats (194–288 g) subjected to a 9‐week CUMS protocol. Anxiety‐like behaviors were evaluated using the EPM, with open arm time serving as the principal outcome measure. In the ketamine‐treated group, open arm times remained below 50 s, while in the ketamine‐treated stress group, open arm times increased to approximately 100 s, indicating partial anxiolytic efficacy. Similarly, Pei et al. (2025) applied a 6‐week CUMS paradigm followed by repetitive transcranial magnetic stimulation (rTMS) in male Sprague‐Dawley rats (170–200 g; 5–6 weeks old). Open arm times were less than 50 s in stressed animals, increased to over 100 s in the treatment group, and ranged between 50 and 100 s in controls, collectively demonstrating that therapeutic interventions alleviate anxiety‐like phenotypes. Previous studies in which fecal bolus counts provided important information about anxiety and stress were mostly obtained in open field tests (Haider et al. 2014; Sadegzadeh et al. 2020; Sturman et al. 2018). Walf and Frye (2007) used the amount of fecal boli to measure anxiety levels in the EPM test. We also measured the defecation, or fecal bolis count after the EPM test. The amount of fecal bolis was higher in the CUMS group than in the control groups. However, there were fewer fecal boli in the CUMS group receiving chronic quetiapine, suggesting a significant reduction in stress‐ and anxiety‐like responses. In our study, anxiety‐like behaviors were quantified based on closed arm occupancy, with treatment group animals exhibiting significantly reduced closed arm times (35.10 ± 14.61 s) compared to controls (93.00 ± 38.99 s), reflecting an anxiolytic effect. Although open arm time and closed arm time were inversely related behavioral indices, both approaches capture the same underlying construct of anxiety regulation. As such, despite differences in reporting metrics, the observed reduction in closed arm time in our study is broadly consistent with the increased open arm occupancy reported by Kingir et al. (2023) and Pei et al., (2025) collectively supporting the anxiolytic potential of the interventions.

While several studies have demonstrated the antidepressant effects of quetiapine in chronic stress models—often in combination with selective serotonin reuptake inhibitors (SSRIs)—evidence supporting quetiapine monotherapy in reversing well‐established stress‐induced behavioral phenotypes remains relatively limited. For example, Orsetti et al. (2007) reported that quetiapine at 2 mg/kg/day prevented the onset of anhedonia in male rats exposed to a 6‐week chronic mild stress (CMS) paradigm, with sucrose preference levels exceeding 80% by the fifth week. More recently, Grolli et al. (2023) demonstrated that quetiapine monotherapy at 20 mg/kg/day significantly reduced immobility time in the forced swim test and reversed CMS‐induced oxidative stress and inflammatory changes, highlighting its multi‐dimensional efficacy. Complementing these findings, Moreines et al. (2017) showed that chronic quetiapine treatment (10 mg/kg/day for 21 days) selectively restored dopaminergic neuron firing in the ventral tegmental area of CMS‐exposed rodents, but not in controls—providing mechanistic support for stress‐specific neuroadaptive effects. Our study builds upon this emerging evidence by demonstrating that quetiapine monotherapy at 10 mg/kg/day for 30 days reverses chronic stress‐induced anxiety‐ and depression‐like phenotypes, including anhedonia, behavioral despair, and avoidance behavior in female Wistar albino rats. Unlike prior research that primarily emphasized prophylactic or adjunctive treatment approaches, our findings highlight the therapeutic efficacy of quetiapine as a standalone agent in restoring affective function under sustained stress conditions. These results extend current knowledge by situating behavioral improvements within a translationally relevant framework and further underscore the necessity of dose‐ and time‐dependent considerations in antipsychotic monotherapy for mood disorders.

Furthermore, given that the anxiolytic efficacy of antipsychotics may be influenced by multiple experimental factors—including species, sex, dosage, treatment duration, and the nature of the stress paradigm employed—our findings highlight the necessity for mechanistic exploration in future research. The present findings help to address this gap by providing novel preclinical evidence for the therapeutic potential of quetiapine in the modulation of anxiety‐like behaviors under chronic stress conditions.

The influence of the estrous cycle on affective behaviors in female rodents is a well‐established variable in neurobehavioral research. Numerous studies have demonstrated that hormonal fluctuations across the four estrous stages—proestrus, estrus, metestrus, and diestrus—can modulate behavioral responses in paradigms measuring depression‐ and anxiety‐like phenotypes. Elevated estrogen levels during proestrus, for example, have been linked to decreased immobility in the forced swim test (FST) and increased time in the open arms of the elevated plus maze (EPM), indicative of reduced depressive and anxiety‐like behavior (Walf and Frye 2006; Carrier et al., 2015). Specifically, in the context of chronic unpredictable mild stress (CUMS) models, several preclinical studies have addressed the estrous cycle's interaction with stress vulnerability and behavioral outcomes. For instance, Kokras et al. (2012) showed that the effects of chronic stress on sucrose preference and forced swim immobility differ across estrous stages, with estrus and proestrus conferring partial behavioral resilience compared to diestrus. Similarly, Marcondes et al. (2001) observed that female rats in proestrus exhibited enhanced exploratory behavior and reduced anxiety in EPM compared to those in diestrus.

Importantly, many CUMS studies in female rodents do not synchronize or monitor estrous stages but instead rely on random cycle distribution, citing population‐level averaging across hormonal states as an acceptable trade‐off for translational relevance (Gobinath et al. 2015). Moreover, female rodents subjected to long‐term CUMS often exhibit cycle disruption or prolongation of diestrus due to stress‐induced suppression of the hypothalamic–pituitary–gonadal (HPG) axis, thereby reducing intra‐group hormonal variance (LaPlant et al. 2009). From a translational standpoint, the relevance of sex hormones in mood disorders is echoed in clinical research with human females, where menstrual cycle phase and hormonal contraceptive use have been shown to affect stress perception, cognitive flexibility, and susceptibility to mood dysregulation (Sundström Poromaa, and Gingnell 2014). Thus, while the estrous cycle is a biologically relevant variable, its dynamic nature—and the stress‐induced blunting of cyclicity—must be contextualized within the broader framework of stress models and neuropsychopharmacological interventions. Despite the strengths of this study, several limitations should be acknowledged. Foremost, the experimental design relied exclusively on behavioral assessments without incorporating complementary biochemical or molecular analyses. This absence restricts the interpretative depth regarding underlying neurobiological mechanisms. Specifically, the failure to evaluate key stress‐related biomarkers—such as corticosterone, brain‐derived neurotrophic factor (BDNF), pro‐inflammatory cytokines (e.g., IL‐6, TNF‐α), serotonin levels, and mesolimbic dopamine release—limits the mechanistic understanding of the observed behavioral outcomes. These neurochemical parameters are critical for elucidating the pathways through which antipsychotic agents exert their effects on affective and cognitive functions. Therefore, future studies are warranted to integrate molecular profiling alongside behavioral testing to more comprehensively characterize the neurobiological effects of pharmacological interventions. Although only female rats were employed, estrous cycle monitoring was not conducted during the experimental procedures. Given that fluctuations in ovarian hormones—particularly estrogen and progesterone—can significantly influence affective behaviors in rodents, the absence of estrous cycle tracking may have introduced variability in behavioral readouts. This is particularly relevant for tests such as the forced swim test (FST), elevated plus maze (EPM), and sucrose preference test (SPT), all of which are sensitive to hormone‐mediated neuromodulation. Previous studies have shown that female rats in the proestrus or estrus stages exhibit reduced depressive and anxiety‐like behaviors, whereas those in diestrus display heightened vulnerability (Walf and Frye 2006; Kokras et al. 2012). Nevertheless, it is worth noting that chronic stress paradigms such as CUMS can induce estrous cycle irregularities, including cycle arrest and prolonged diestrus, effectively minimizing within‐group hormonal fluctuations (LaPlant et al. 2009). Additionally, many prior CUMS studies in female rodents have not synchronized estrous cycles but have instead relied on randomized cycle distribution across groups, assuming hormonal variability averages out across large samples (Gobinath et al. 2015). Nonetheless, future investigations would benefit from incorporating vaginal smear analysis or hormonal profiling to better delineate the impact of estrous status on behavioral outcomes and pharmacological responsiveness. Such refinements would further enhance the translational validity of stress models in female subjects, particularly in light of sex‐specific vulnerabilities observed in clinical populations (Sundström Poromaa, and Gingnell 2014). Moreover, recent preclinical evidence suggests that chronic quetiapine administration may influence estrous cyclicity in female rodents. In Sprague‐Dawley rats, oral administration of quetiapine at doses of 10 to 50 mg/kg has been associated with increased irregularities in the estrous cycle, whereas no significant effects were observed at 1 mg/kg (FDA 2004). Similarly, in an 8‐week experimental study involving female Wistar albino rats, quetiapine administered at 10 mg/kg resulted in delayed progression through the proestrus and estrus phases in approximately 25% of animals, though these alterations did not reach statistical significance. However, a significant reduction in ovarian estrogen receptor‐α (E2Rα) immunoreactivity was reported, suggesting possible long‐term endocrine disruption (He et al. 2018). While there are limited clinical data specifically examining quetiapine's impact on the menstrual cycle in women, antipsychotics, including quetiapine, are known to induce hyperprolactinemia, which can lead to menstrual disturbances, amenorrhea, and reduced estrogen levels in female patients (Dickson and Glazer 1999; Inder and Castle 2011). Therefore, the absence of estrous cycle tracking in the present study represents a notable limitation. It is possible that quetiapine itself may have influenced hormonal homeostasis, thereby confounding behavioral outcomes that are sensitive to ovarian steroid fluctuations. Future studies should incorporate vaginal smear cytology or serum hormone profiling to account for estrous phase–dependent variability and to clarify the interaction between antipsychotic treatment and reproductive endocrinology in female models.

## 5 Conclusion

In conclusion, the present study demonstrates that quetiapine exerts significant therapeutic effects in alleviating both depression‐ and anxiety‐like behaviors induced by CUMS. Importantly, these findings underscore the potential utility of quetiapine not only as an adjunctive agent in treatment‐resistant populations but also as a viable first‐line intervention in early‐stage mood disorders. This expands the therapeutic scope of quetiapine beyond its conventional positioning and suggests its broader applicability in stress‐related psychopathologies. Nevertheless, these promising preclinical outcomes warrant further validation through comprehensive investigations across diverse stress paradigms, sex differences, and mechanistic pathways. Future studies incorporating dose‐response analyses, extended treatment durations, and comparative evaluations with other second‐generation antipsychotics will be critical for optimizing treatment strategies. Collectively, our findings provide important translational insights and lay the groundwork for the development of novel, targeted therapeutic approaches aimed at mitigating the complex neurobehavioral consequences of chronic stress exposure.

## Author Contributions

**Esra Komanovalı**: methodology, conceptualization, resources, data curation. **Burcu Çevreli**: investigation, writing – original draft, writing – review and editing, project administration, supervision, methodology, validation, formal analysis, software, data curation, resources, visualization, funding acquisition, conceptualization. **Öznur Özge Özcan**: conceptualization, investigation, funding acquisition, writing – original draft, methodology, validation, visualization, writing – review and editing, software, formal analysis, project administration, data curation, supervision, resources.

## Conflicts of Interest

The authors declare no conflicts of interest.

## Peer Review

The peer review history for this article is available at https://publons.com/publon/10.1002/brb3.70755

## Data Availability

The data that support the findings of this study are available on request from the corresponding author. The data are not publicly available due to privacy or ethical restrictions
